# Detection of Splenic Tissue Using ^99m^Tc-Labelled Denatured Red Blood Cells Scintigraphy—A Quantitative Single Center Analysis

**DOI:** 10.3390/diagnostics12020486

**Published:** 2022-02-14

**Authors:** Adrien Holzgreve, Friederike Völter, Astrid Delker, Wolfgang G. Kunz, Matthias P. Fabritius, Matthias Brendel, Nathalie L. Albert, Peter Bartenstein, Marcus Unterrainer, Lena M. Unterrainer

**Affiliations:** 1 Department of Nuclear Medicine, University Hospital, LMU Munich, 81377 Munich, Germany; friederike.voelter@med.uni-muenchen.de (F.V.); astrid.gosewisch@med.uni-muenchen.de (A.D.); matthias.brendel@med.uni-muenchen.de (M.B.); nathalie.albert@med.uni-muenchen.de (N.L.A.); peter.bartenstein@med.uni-muenchen.de (P.B.); lena.unterrainer@med.uni-muenchen.de (L.M.U.); 2 Department of Radiology, University Hospital, LMU Munich, 81377 Munich, Germany; wolfgang.kunz@med.uni-muenchen.de (W.G.K.); matthias.fabritius@med.uni-muenchen.de (M.P.F.); marcus.unterrainer@med.uni-muenchen.de (M.U.)

**Keywords:** selective spleen scintigraphy, RBC, splenosis, splenule, accessory spleen, peritoneal carcinomatosis

## Abstract

Background: Red blood cells (RBC) scintigraphy can be used not only for detection of bleeding sites, but also of spleen tissue. However, there is no established quantitative readout. Therefore, we investigated uptake in suspected splenic lesions in direct quantitative correlation to sites of physiologic uptake in order to objectify the readout. Methods: 20 patients with Tc-99m-labelled RBC scintigraphy and SPECT/low-dose CT for assessment of suspected splenic tissue were included. Lesions were rated as vital splenic or non-splenic tissue, and uptake and physiologic uptake of bone marrow, pancreas, and spleen were then quantified using a volume-of-interest based approach. Hepatic uptake served as a reference. Results: The median uptake ratio was significantly higher in splenic (2.82 (range, 0.58–24.10), *n* = 47) compared to other lesions (0.49 (0.01–0.83), *n* = 7), *p* < 0.001, and 5 lesions were newly discovered. The median pancreatic uptake was 0.09 (range 0.03–0.67), bone marrow 0.17 (0.03–0.45), and orthotopic spleen 14.45 (3.04–29.82). Compared to orthotopic spleens, the pancreas showed lowest uptake (0.09 vs. 14.45, *p* = 0.004). Based on pancreatic uptake we defined a cutoff (0.75) to distinguish splenic from other tissues. Conclusion: As the uptake in extra-splenic regions is invariably low compared to splenules, it can be used as comparator for evaluating suspected splenic tissues.

## 1. Introduction

Scintigraphy with administration of technetium-99m (^99m^Tc)-labelled denatured red blood cells (RBCs) is a commonly applied tool for detection of unclear bleeding sites. However, due to physiological RBC molting, RBC scintigraphy could also be used for detection of ectopic spleen tissue, splenules, or following splenic trauma or surgery [1]: Selective spleen scintigraphy with intravascular administration of ^99m^Tc-labelled RBCs is indicated for assessing size, shape, and position of the spleen, detecting and measuring spleen masses, identifying functioning splenic tissue, as well as evaluating suspected functional asplenia [2,3,4]. Compared to spleen detection using hepatobiliary scintigraphy or technetium-99m-sulfur colloid, the selective spleen scintigraphy with RBCs has a higher diagnostic value due to the absence of liver uptake and its increased specificity [2,5,6,7].

Accidental implantation of splenic tissue (splenosis) could be demonstrated in a reliable way by selective spleen scintigraphy, especially with a concomitant SPECT [8,9]. Experimental studies could demonstrate that splenic tissue can survive and grow when transplanted to abnormal sites [10]. In this context, splenosis is a frequent finding after trauma to the spleen with rupture of the splenic capsule, with an incidence of 16–67% [1,9,11], and can be defined as the heterotopic auto transplantation of splenic tissue onto the peritoneal surfaces, in the splenic fossa, in the gastrointestinal tract, the liver, the subcutaneous region and, rarely, in the lungs or pleural space if splenic rupture is associated with rupture of the diaphragm [9,11]. There are even cases known of heterotopic spleen tissue in the head after an accident [10,11]. Patients with splenosis are often asymptomatic but, for example, intraabdominal lesions may stimulate adhesions which may lead to intestinal obstruction [9]. In this context, splenosis can be mistaken for endometriosis, intrathoracic neoplasm, or angioma, but also for primary intraabdominal tumor [12,13]. Splenosis has indeed been reported to mimic tumor recurrence in patients with known prior tumors and can also mimic metastatic peritoneal spread of a known tumor by means of peritoneal carcinomatosis [14,15,16], a diagnosis which can lead to a fulminant change of oncological treatment [17,18]. Despite this major clinical impact, splenosis remains diagnostically challenging in conventional imaging. Thus, RBC scintigraphy could be of particular relevance for the evaluation of suspected splenic tissue and for subsequently guiding patient management. However, there is no established quantitative readout for rating suspected splenic lesions as either vital splenic tissue or as other tissues, such as metastases, in RBC scintigraphy. Hence, until now clinical decision-making is mainly based on individual reader-dependent qualitative assessment.

Therefore, in this study, we demonstrate RBC scintigraphy using a newly defined quantitative cutoff as a highly feasible method for the detection of splenic tissues.

## 2. Materials and Methods

### 2.1. Study Design and Inclusion Criteria

This retrospective analysis was approved by the institutional ethics committee of the LMU Munich (# 21-0325). Patients who received a ^99m^Tc-labelled denatured red blood cells scintigraphy with available concomitant single photon emission tomography (SPECT)/low dose computed tomography (ldCT) for evaluation of suspected splenic tissue from 2013 to 2021 were included. All patients gave written informed consent prior to scintigraphy and concomitant SPECT/ldCT as part of the clinical routine.

First, uptake in sites of physiologic uptake was quantified. Second, uptake in suspected splenic lesions was quantified and compared to uptake in sites of physiologic uptake. Finally, a potential quantitative cutoff for the differentiation of vital splenic tissue from other tissues was evaluated based on the previously performed measurements; here, a series of unequivocal cases with, if available, histology or otherwise imaging/clinical follow up served as control.

### 2.2. Radiopharmaceutical and Imaging Protocol

For evaluation of suspected splenic tissue, a median of activity of 148 MBq (Q1–Q3 interquartile range, 140–153 MBq) technetium-99m heat damaged RBCs were prepared as described previously [4] and administered intravenously. SPECT and planar imaging were performed on a dual-headed Siemens Symbia T2 SPECT/CT or a Siemens Symbia Intevo T16 SPECT/CT system (Siemens Healthineers, Erlangen, Germany), using a low-energy high-resolution collimator. Planar images of the abdomen were acquired over 10 min. SPECT/low dose CT scan was initiated 30 min after tracer injection. SPECT acquisition parameters were 32 projections per head with 25 s per projection and a projection matrix of 128 × 128 pixels (4.7952 × 4.7952 mm^2^). SPECT reconstruction was performed via Hermes (Hermes Medical Solutions, Stockholm, Sweden) using the OSEM algorithm (3 iterations and 16 subsets) with Gaussian post-filtering of 1.10 cm full-width-half-maximum and included CT-based attenuation correction as well as resolution compensation. For further evaluation, SPECT images were transferred to a Hermes workstation.

### 2.3. Data Analysis

**Figure 1:**
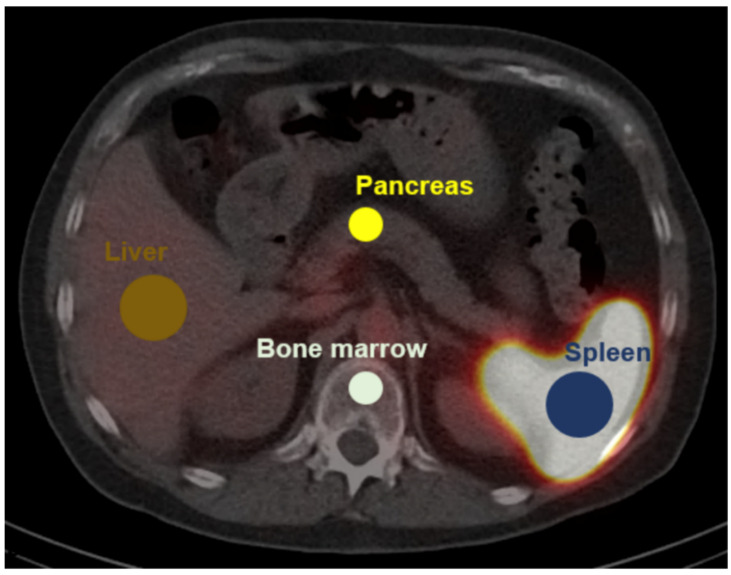
VOI definition for quantification of physiologic uptake. ^99m^Tc-labelled denatured red blood cells single photon emission tomography (SPECT) is fused on low dose computed tomography (CT). Diameter of spherical 3D-VOIs: pancreas 1.5 cm, bone marrow 1.5 cm, spleen 3.0 cm, liver 3.0 cm.

For uptake quantification, hepatic uptake served as reference tissue and was quantified using a spherical 3.0 cm diameter volume-of-interest (VOI) in the non-diseased right hepatic lobe [19]. Uptake in sites of physiologic uptake was quantified using spherical VOIs (diameter 1.5 cm: bone marrow and, if available, pancreas; diameter 3.0 cm: orthotopic spleen [20]). Figure 1 illustrates the VOI definition. For evaluation of suspected splenic tissue, scans were reviewed by 4 nuclear medicine physicians and rated either as vital splenic or non-splenic lesions. The uptake characteristics of the lesions were then quantified using a VOI-based approach consisting of a manually drawn VOI in three adjacent axial layers centered on the maximum uptake of the lesion. Based on uptake quantification in sites of physiologic uptake, a quantitative cutoff for the differentiation of splenic from non-splenic tissue was defined.

### 2.4. Data Statistics

SPSS for Windows (version 25.0; SPSS, Chicago, IL, USA) was used for statistical analyses. Normal distribution was assessed using the Shapiro–Wilk test. The Wilcoxon signed-rank test was used to compare dependent and not-normally distributed continuous parameters. The unpaired Mann–Whitney U test was used to compare independent and not-normally distributed continuous parameters. Statistical significance was defined as a two-tailed *p*-value < 0.05.

## 3. Results

### 3.1. Patient Characteristics

20 patients were included in the study. Characteristics of patients scanned for the evaluation of suspected splenic tissue are shown in Table 1. One patient was excluded from quantitative analyses due to outlying low applied radioactivity (#18; 77 MBq).

### 3.2. Uptake Quantification

#### 3.2.1. Sites of Physiologic Uptake

**Figure 2:**
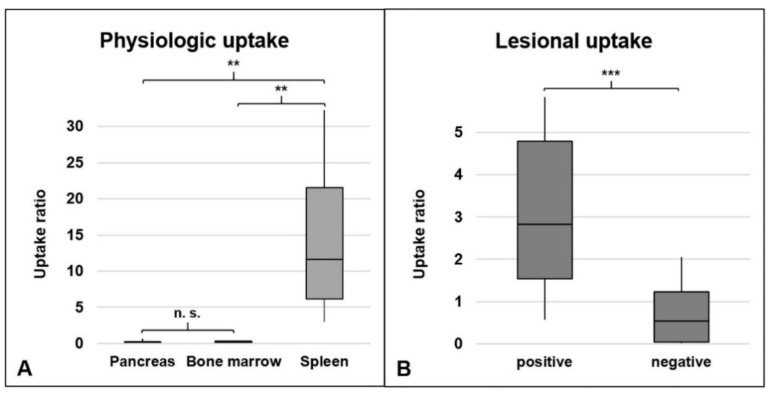
Uptake quantification in RBC scintigraphy in the overall group. (**A**) = Uptake in sites of physiologic uptake. (**B**) = Uptake in suspected splenic lesions. Referenced to liver uptake (uptake ratio). Positive = consensually rated as splenic tissue. Negative = consensually rated as non-splenic tissue. n.s. = not significant. ** *p* < 0.01, *** *p* < 0.001.

The median ratio of physiologic uptake in the pancreas was 0.09 (range 0.03–0.67), in the bone marrow 0.17 (range 0.03–0.45), and in the orthotopic spleen 14.45 (range 3.04–29.82). Compared to orthotopic spleen tissues, the pancreas showed the lowest uptake characteristics (median 0.09 vs. 14.45, *p* = 0.004), followed by the bone marrow (median 0.17 vs. 14.45, *p* = 0.002). The uptake characteristics in bone marrow and pancreas were rather similarly low (median 0.09 vs. 0.17, *p* = 0.261), see Figure 2A. All values for physiologic uptake are shown in Table 1.

#### 3.2.2. Suspected Splenic Lesions

55 abdominal lesions were investigated. One lesion was excluded from quantitative analyses due to spill-in from the adjacent orthotopic spleen. The number of lesions per patient rated as either positive or negative for splenic tissue in RBC scintigraphy/SPECT are displayed in Table 1. Median VOI size for quantification of suspected splenic lesions was 1.32 mL (range 0.33–13.45 mL). The median uptake ratio was significantly higher in splenic lesions (2.82 (range, 0.58–24.10), *n* = 47) compared to other lesions (0.49 (range, 0.01–0.83), *n* = 7), *p* < 0.001. The uptake quantification of positive and negative lesions is shown in Figure 2B.

Interestingly, at least 5 new, priorly unnoted lesions were discovered due to increased uptake in RBC scintigraphy (see Table 1).

### 3.3. Evaluation of a Potential Quantitative Cutoff for Clinical Reading

#### 3.3.1. Definition of a Quantitative Cutoff in Relation to Physiologic Uptake in a Subgroup of Unequivocal Cases

**Figure 3:**
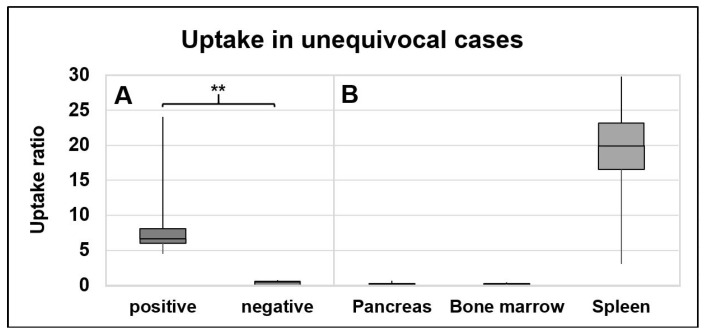
Uptake quantification in RBC scintigraphy in unequivocal cases. (**A**) = Uptake in suspected splenic lesions. (**B**) = Uptake in sites of physiologic uptake. Referenced to liver uptake (uptake ratio). Positive = consensually rated as splenic tissue. Negative = consensually rated as non-splenic tissue. ** *p* < 0.01.

First, only the cases with unequivocal results based on uptake intensity in clinical reading (*n* = 5 with clearly suspected splenic tissue and *n* = 5 with clearly suspected metastases or other non-splenic tissues) and additionally with, if available, histological or otherwise imaging or clinical follow up for confirmation of clinical reading results were selected (*n* = 3 histology, *n* = 3 ultrasonography follow up, *n* = 1 PET/CT follow up, *n* = 3 clinical follow up of each >18 months). These cases showed even more distinctly than in the overall group (see Section 3.2.2) that the median uptake ratio was significantly higher in splenic lesions (8.10 (range, 4.50–24.10)) as compared to other lesions (0.49 (range, 0.01–0.74)), *p* = 0.004, see Figure 3A. In lesions rated as spleen tissue, the median uptake ratio was higher as compared to uptake characteristics in the pancreas (0.19 (range, 0.03–0.67), *p* = 0.063) and bone marrow (0.22 (range, 0.03–0.45), *p* = 0.031), but still somewhat lower than in the orthotopic spleen (19.95 (range, 3.04–29.32), *p* = 0.500), see Figure 3A,B. In lesions rated as non-splenic tissue, the median uptake ratio was rather similar compared to the pancreas (0.19 (range, 0.03–0.67), *p* = 0.156) and the bone marrow (0.22 (range, 0.03–0.45), *p* = 0.156), but lower than in the orthotopic spleen (19.95 (range, 3.04–29.32), *p* = 0.125), see Figure 3A,B.

With regard to the inferior uptake intensity of suspected splenic lesions in comparison to the orthotopic spleens, approaches using an objective cutoff based on physiologic uptake in the orthotopic spleen were rejected for further analyses. Yet, the pancreas showed lowest uptake characteristics, with clearly inferior uptake as compared to positive lesions (median 0.19 vs. 8.10 vs., *p* = 0.063) and even slightly inferior uptake as compared to the bone marrow (0.19 vs. 0.22, *p* = 0.469). Therefore, the following quantitative cutoff defined in relation to the pancreatic uptake is proposed for the differentiation of splenic from non-splenic lesions:

**Cutoff** = (median uptake ratio in pancreas + 2 × SD) = (0.19 + 2 × 0.28) = **0.75** (1)

#### 3.3.2. Proof-of-Principle Quantitative Reading in All Cases in Direct Comparison to Standard Qualitative Clinical Reading

**Figure 4:**
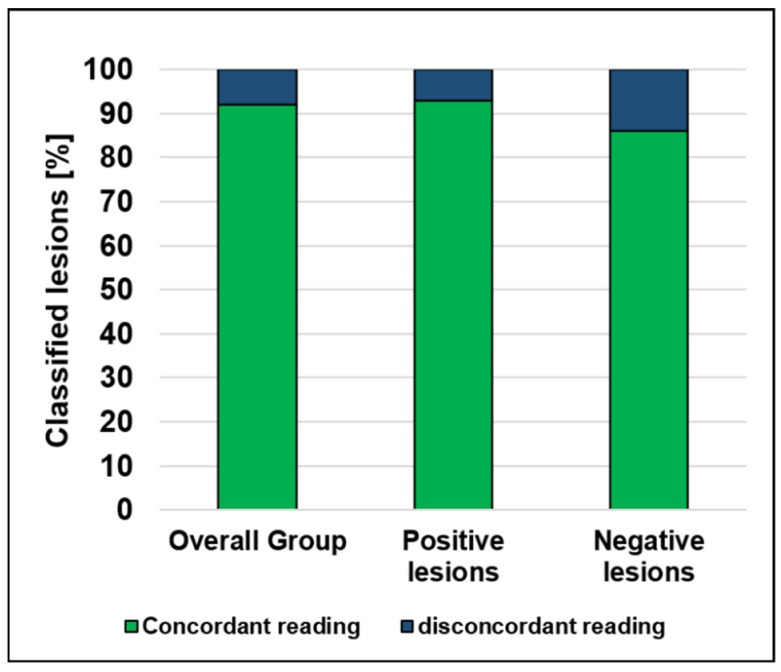
Performance of quantitative reading in direct comparison to standard qualitative clinical reading.

When using the objective reader-independent cutoff based on physiologic pancreatic uptake as proposed above in all cases included in the quantitative analysis, in 50 out of 54 cases the lesions were concordantly classified with regard to standard clinical reading. For the positive lesions, 44 out of 47 were concordantly classified with the clinical reading, and 6 out of 7 negative lesions were concordantly classified with the clinical reading, see Figure 4.

#### 3.3.3. Exemplary Use of the Quantitative Cutoff in Histology-Proven Cases

To illustrate the benefit of RBC scintigraphy using a quantitative cutoff for the differentiation of splenic from non-splenic lesions in clinical routine, a case of negative reading (i.e., lesional uptake below the cutoff), a case of positive reading (i.e., with lesional uptake above the cutoff) as well as two challenging cases in which the cutoff helped the clinical reading are presented.

**Figure 5:**
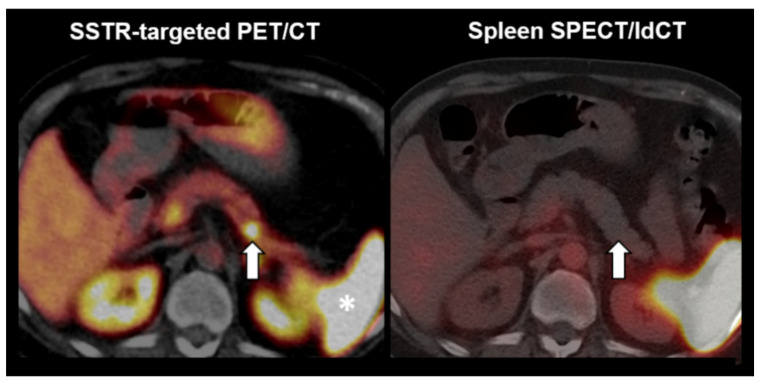
Example of a negative reading in spleen scintigraphy. (**Left**) Somatostatin receptor (SSTR)-targeted PET/CT. (**Right**) ^99m^Tc-labelled denatured red blood cells SPECT fused to low dose CT. Arrows = the suspected lesion. Asterisk = physiologic SSTR radioligand uptake in the spleen.

Figure 5 illustrates the case of a lesion rated as non-splenic tissue. A 67-year-old woman with an incidental lesion in the pancreatic tail presented for somatostatin receptor (SSTR)-targeted PET/CT (left). A strong focal tracer uptake in PET/CT suggested a neuroendocrine pancreatic neoplasm (see left arrow). The latter, however, cannot be differentiated from an intrapancreatic splenule in PET/CT, since both entities display a comparably high SSTR-ligand uptake despite their diametrically opposed prognosis (note the high physiologic SSTR-ligand uptake in the spleen; see asterisk). Yet, the pancreatic lesion was not visible in endoscopy and, therefore, not accessible to ultrasound-guided fine needle aspiration cytology and biopsy. Hence, the interdisciplinary tumor board review recommended ^99m^Tc-labelled denatured red blood cells scintigraphy to rule out a splenule (right; SPECT fused to low dose CT). Here, no increased uptake is noted in the pancreatic lesion (see right arrow). Taking into account the scintigraphy results, a laparoscopic distal pancreatectomy was performed, and histopathology ultimately confirmed the diagnosis of a neuroendocrine tumor of the pancreas. Using the quantitative reading in selective spleen scintigraphy, the lesion would have been correctly classified (lower lesional uptake ratio than the cutoff, i.e., 0.49 < 0.75).

**Figure 6:**
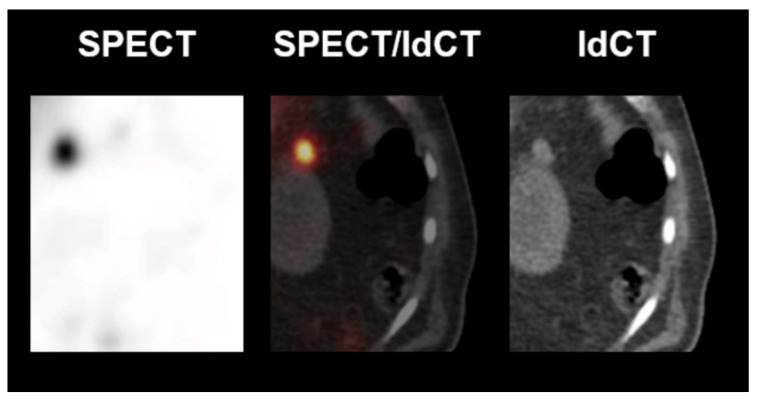
Example of a positive reading in spleen scintigraphy. Left anterior perigastric lesion. (**Left**) ^99m^Tc-labelled denatured red blood cells SPECT. (**Middle**) ^99m^Tc-labelled denatured red blood cells SPECT fused to low dose CT. (**Right**) low dose CT.

Figure 6 illustrates the case of a lesion rated as splenic tissue. A 63-year-old man with newly diagnosed esophageal adenocarcinoma presented with multiple unclear abdominal lesions. The patient had experienced traumatic rupture of the spleen several years before. ^99m^Tc-labelled denatured red blood cells scintigraphy was performed (SPECT fused to low dose CT) in order to differentiate splenosis secondary to rupture of the spleen from potential lymph node metastases and peritoneal carcinomatosis secondary to the esophageal carcinoma. The lesions showed pronounced tracer uptake and, therefore, suggested splenic tissue (e.g., see perigastric lesion in the figure). Hence, after 3 neoadjuvant cycles of fluorouracil plus leucovorin, oxaliplatin, and docetaxel (FLOT) chemotherapy, a thoracoabdominal partial esophagectomy with total gastrectomy and locoregional lymphadenectomy was performed in curative intention. Histopathology showed no evidence of lymph node metastases or peritoneal carcinomatosis (ypT0, ypN0 (0/34), L0, V0, Pn0, UICC-Stadium 0, locally R0). Using the quantitative reading in selective spleen scintigraphy, the lesion would have been correctly classified (higher lesional uptake ratio than the cutoff, i.e., 1.22 > 0.75).

**Figure 7:**
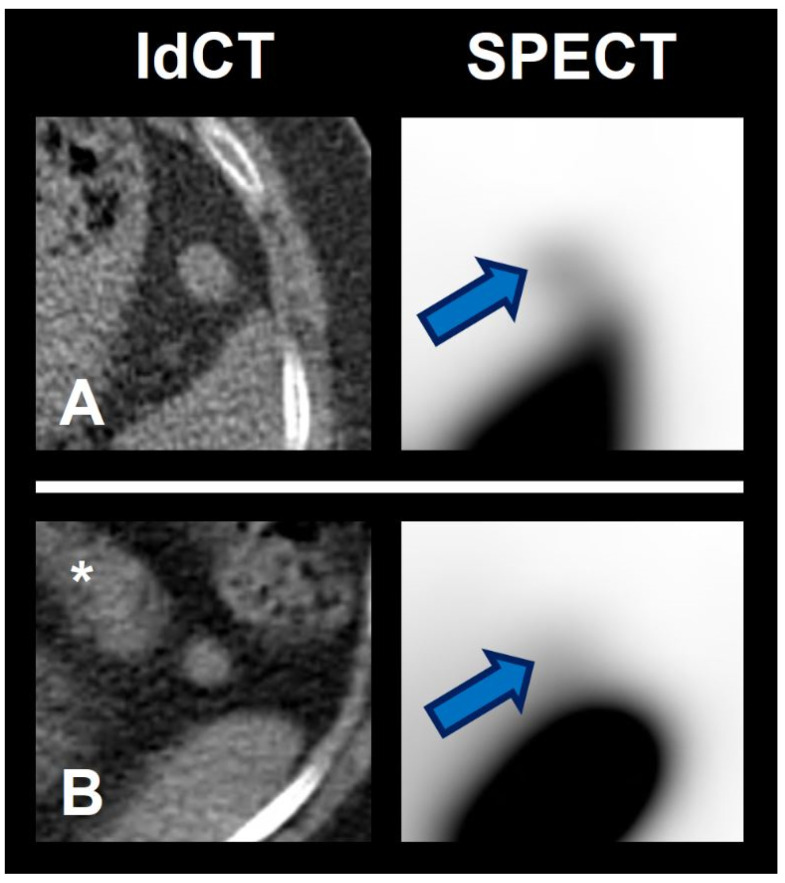
Example of two challenging cases in spleen scintigraphy reading. (**Left**) low dose CT. (**Right**) ^99m^Tc-labelled denatured red blood cells SPECT. (**A**) = Rated as spleen tissue using the quantitative cutoff. Clinical follow up showed no evidence of a progression of the perisplenic lesion. (**B**) = Not rated as spleen tissue using the quantitative cutoff. Endosonographic biopsy was performed and confirmed the rating. Arrows = lesional uptake. Asterisk = pancreas.

Figure 7 illustrates two challenging cases with visually similar uptake in spleen scintigraphy. In both cases, the use of the newly proposed quantitative cutoff approach would have led to the correct diagnosis and, therefore, would have helped significantly with the unclear visual findings in clinical reading. Figure 7A shows the scan of 60-year-old woman referred for spleen scintigraphy due to an incidental, unclear perisplenic lesion. Using merely visual reading, the lesion would have been prone to be classified as negative, as it has a significantly lower uptake than the nearby orthotopic spleen. Using the cutoff, however, the lesion would have been correctly rated as positive with a ratio of 6.0 (clearly > 0.75). Clinical follow up >18 months showed no evidence of a progression of the perisplenic lesion. Figure 7B shows the scan of a 56-year-old woman with an unclear lesion near the pancreatic tail. Although displaying minimally lower uptake in visual reading as compared to the lesion in Figure 7A, there is still more uptake than in a clearly negative case (e.g., Figure 5) and, therefore, the lesion would have been at risk of being classified as positive in standard clinical reading. Using the newly proposed quantitative cutoff approach, however, the lesion would have been correctly rated as negative (ratio < 0.75). In this case, an endosonography was performed and the histopathological findings were most compatible with a partially sclerosed lymph node.

## 4. Discussion

Splenosis is the heterotopic autotransplantation of splenic tissue following splenic trauma or splenectomy and is mostly found in the peritoneal, pelvic, or even thoracic cavity. The incidence of splenosis varies from 26–65% in patients with traumatic splenic rupture [21,22]. Heterotopic splenic tissue is diagnostically challenging, as it can easily be mistaken as metastatic peritoneal spread in conventional imaging, especially in patients with a known tumoral disease, and could, therefore, lead to unnecessary surgeries or other procedures with potential harm to the patient. Radiolabeled heat-damaged red blood cells (RBC) scintigraphy is a reliable and non-invasive imaging method to confirm the presence of splenic tissue [1]. However, until now there has been no defined standard quantitative approach to clinical reading of RBC scintigraphy.

Therefore, to distinguish between splenic tissue and metastatic abdominal sites in inconclusive cases in the future, we conducted a single-center analysis investigating the uptake characteristics in suspected splenic lesions in direct quantitative correlation to physiologic uptake in sites of physiologic uptake. To the best of our knowledge, this is the first study, which describes a visual and quantitative method to identify splenic tissue in relation to the surrounding tissues.

Compared to other lesions, splenic lesions showed a significantly higher uptake ratio in RBC scintigraphy. However, interestingly, suspected splenic lesions had an inferior uptake compared to orthotopic spleens (e.g., see Figure 3A,B). Therefore, due to the quantitatively inferior uptake intensity of suspected splenic lesions in comparison to the orthotopic spleens, approaches using an objective cutoff based on physiologic uptake in the orthotopic spleen were rejected for further analyses. The cause of this differential uptake remains unclear; one might speculate that a partial volume effect is a contributor to this phenomenon (the median VOI size for quantification of suspected splenic lesions was low with only 1.32 mL and a range of 0.33–13.45 mL). In addition, the range of uptake in orthotopic spleens was rather high (3.04–29.32, see error bars in Figure 3). Instead, the uptake characteristics in the pancreas and bone marrow seemed to be more suitable for the definition of an objective quantitative cutoff, as they were (1) comparably low in all cases (low range of 0.03–0.67 and 0.03–0.45, respectively); (2) similarly low as compared to the uptake in negative lesions (range, 0.01–0.87); and (3) invariably lower than the uptake in positive lesions (median 8.10 vs. 0.19, *p* = 0.063; see Figure 3A,B). As the pancreatic uptake proved to be even slightly lower than the uptake in the bone marrow (0.19 vs. 0.22, *p* = 0.469), and as intrapancreatic splenules vs. metastases are an important clinical differential diagnosis to be addressed with RBC scintigraphy, uptake characteristics of the pancreas were eventually used for definition of the cutoff. Thus, we proposed a quantitative cutoff solely based on the physiologic uptake of the pancreas (and not just a cutoff centered in between the latter and the orthotopic spleen uptake) for the differentiation of splenic from non-splenic lesions, i.e., 0.75. This cutoff was established in a subgroup of unequivocal cases with, if available, histology or imaging/clinical follow up for control.

The proof-of-principle application of this cutoff showed a concordant classification of the corresponding lesions in 92.6% of cases with regard to standard clinical reading. To illustrate the potential application of the cutoff in clinical routine, illustrative cases with available histology were presented. Here, applying this cutoff could indeed exclude/confirm a splenule, with subsequent clinical benefit, e.g., in one case it supported the suspected diagnosis of an intrapancreatic neoplasia and subsequently lead to the decision to perform a distal pancreatectomy. Histopathology ultimately confirmed the diagnosis of a neuroendocrine tumor of the pancreas and not of a splenule, as correctly predicted by RBC scintigraphy. Of note, the newly proposed method using a cutoff may especially support decision making in challenging cases which would potentially have been misclassified using standard visual reading alone (see Figure 7), illustrating the presumable added value of quantitative reading in clinical routine.

Limitations of this study arise from the rather small sample size and the merely internal validation in a single institution setting, which do not impose a more general applicability of the established method per se. Yet it has to be noted, that this is probably the largest number to date of RBC scans quantitatively analyzed in one study for the assessment of splenic tissue, and that future studies can, therefore, relate to and build upon those initial results. Another limitation is the lack of histologic validation for most cases, which is partly due to the retrospective study design, and here mainly to the much higher number of positively rated lesions, which regularly do not result in a biopsy or resection within routine clinical practice. Therefore, although 20 patients were enrolled in total, the quantitative cutoff was established in a subgroup of only 10 patients with, if available, histological or otherwise imaging/clinical follow up. However, further prospective studies for detection of splenic tissues are underway to verify this newly defined cutoff in more histologically verified cases.

This study could show for the first time that using a defined quantitative cutoff in RBC scintigraphy is a feasible method for the assessment of suspected splenic tissue as well as for the detection of previously unknown sites of splenosis. This method could avoid unnecessary biopsies, surgical exploration, further imaging or misleading of oncological procedures.

## Figures and Tables

**Table 1:** Patient characteristics and uptake characteristics. Uptake values are referenced to liver uptake. ^1^ If information not available, lesions were considered as previously known (“0”). Pos. = positive. Neg. = negative. * = one additional lesion excluded due to spill-in from the adjacent spleen. ** = excluded due to exocrine pancreatic insufficiency secondary to cystic fibrosis. M = male. F = female. PPPD = pylorus-preserving pancreaticoduodenectomy. n/a = not available.

Pat. ID	Sex	Age [y]	Splen-Ectomy	Pancrea-Tectomy	Uptake in Sites of Physiologic Uptake			Number of Lesions as Rated in Scintigraphy		Newly Discovered Lesions ^1^
					Bone Marrow	Pancreas	Regular Spleen	Pos.	Neg.	
**1**	m	44	partially	no	0.45	0.25	n/a	9	0	0
**2**	f	67	no	no	0.27	0.27	23.18	1 *	1	2
**3**	m	68	yes	no	0.03	0.03	n/a	1	2	1
**4**	m	51	yes	no	0.22	0.18	n/a	1	1	0
**5**	f	67	no	no	0.09	0.09	14.45	0	0	0
**6**	f	84	yes	distal	0.22	n/a	n/a	4	0	0
**7**	m	67	yes	distal	0.12	n/a	n/a	1	0	0
**8**	m	35	no	no	0.16	0.05	3.91	1	0	0
**9**	f	60	no	no	0.25	0.53	19.95	1	0	0
**10**	m	62	yes	no	0.17	0.15	n/a	3	0	0
**11**	m	62	yes	no	0.15	0.03	n/a	1	0	0
**12**	w	73	no	no	0.08	0.06	3.70	1	0	0
**13**	f	60	no	no	0.15	0.67	29.82	1	0	0
**14**	f	59	yes	distal	0.17	n/a	n/a	1	0	0
**15**	f	39	partially	no	0.20	0.06	n/a	8	0	0
**16**	f	56	no	no	0.07	0.19	16.59	0	1	1
**17**	m	64	no	PPPD	0.19	n/a	3.86	1	1	1
**18**	f	28	no	no	0.12	n/a **	0.61	0	1	0
**19**	m	50	yes	no	0.19	0.04	n/a	12	0	0
**20**	m	51	No	distal	0.22	0.07	3.04	0	1	0

## Data Availability

The numerical data presented in this study are available in Table 1. Further requests can be addressed to the corresponding author.
